# Yes, I Am Ready Now: Differential Effects of Paced versus Unpaced Mating on Anxiety and Central Oxytocin Release in Female Rats

**DOI:** 10.1371/journal.pone.0023599

**Published:** 2011-08-16

**Authors:** Kewir D. Nyuyki, Martin Waldherr, Sandra Baeuml, Inga D. Neumann

**Affiliations:** Department of Behavioral and Molecular Neurobiology, University of Regensburg, Regensburg, Bavaria, Germany; University of Wuerzburg, Germany

## Abstract

Sexual activity and partner intimacy results in several positive consequences in the context of stress-coping, both in males and females, such as reduced state anxiety in male rats after successful mating. However, in female rats, mating is a rewarding experience only when the estrous female is able to control sexual interactions, i.e., under paced-mating conditions. Here, we demonstrate that sex-steroid priming required for female mating is anxiolytic; subsequent sexual activity under paced mating conditions did not disrupt this anxiolytic priming effect, whereas mating under unpaced conditions increased anxiety-related behavior. In primed females, the release of the neuropeptide oxytocin (OT) within the hypothalamic paraventricular nucleus was found to be elevated and to further increase during paced, but not unpaced mating. Central administration of an OT receptor antagonist partly prevented priming/mating-induced anxiolysis indicating the involvement of brain OT in the anxiolysis triggered by priming and/or sexual activity.

These findings reveal that the positive consequences of mating in females are dependent on her ability to control sexual interactions, and that brain OT release is at least in part the underlying neurobiological correlate.

## Introduction

Sexual activity has been shown to exert positive health effects in humans and other mammalian species. In this context, correlations between sexual intercourse and various psychological and physiological parameters have been described in women, such as relationship quality [1], weight gain [2] and stress reactivity [3], with regular couple intimacy reducing basal salivary cortisol levels [4]. In support, in female rodents, sexual activity was found to increase life expectancy [5] and to induce a hedonic state [6], [7]. Although the central mechanisms behind these findings are largely unknown, the neuropeptide oxytocin (OT) is a possible mediator of the positive effects of close social interactions in general [8], and specifically of sexual activity [9]. Brain OT exerts significant anxiolytic and stress-attenuating actions [10]–[12] and rewarding effects [13], [14]. Moreover, OT is also an important regulator of male and female sexual functions [15], [16].

In a previous study, we demonstrated increased OT release within the hypothalamic paraventricular nucleus (PVN) - a region integrating behavioral and neuroendocrine stress responses [17] - during mating in male rats. Such centrally released OT was found to mediate mating-induced anxiolysis up to 4 hrs after successful mating [9]. Whether similar behavioral and neuroendocrine consequences of mating can also be found in females is completely unknown.

In female rats, OT neurons within the PVN are activated during sexual activity [18], [19], and increased OT levels were found in the cerebrospinal fluid in response to vaginocervical stimulation mimicking birth-related conditions [20]. Recently, we have demonstrated increased extracellular concentrations of OT in the nucleus accumbens in a subset of female prairie voles during unrestricted interactions with a male [21]. OT is also secreted into the blood stream during orgasm both in men and women [22].

In general, high activity of the brain OT system, as found for example in the peripartum period [23], has been linked to an attenuated stress responsiveness including reduced anxiety-related behavior [24]. This led us to hypothesize that mating may also activate the brain OT system and reduce the emotional stress response in females. We further hypothesized that such an effect would be dependent on the mating conditions for the female, i.e. paced mating versus unpaced mating. Under semi-naturalistic conditions, ninety percent of intromissions are preceded by female approach behavior [25], demonstrating female control of sexual interactions. The readiness of the female to engage in sexual activity is shown by several proceptive (e.g. solicitation, hopping and darting) and receptive (lordosis) behaviors [26], and endogenous fluctuations of ovarian sex steroids as seen during the estrus cycle are largely involved [27]. Moreover, plasma estrogen and progesterone levels peak during proestrus when state anxiety is lowest [27], [28]. Thus, besides mating conditions, modulatory effects of ovarian hormones must be taken into account in the laboratory when studying mating-induced effects on anxiety-related behavior in ovariectomized steroid-primed female rats.

Under experimental conditions, successful paced mating can be achieved by enabling the female to escape the male [29]. Such an experimental setup prevents the male from dictating the pace of intromissions. While an increased activity of OT-containing neurons within the PVN has been demonstrated during both paced [18] and unpaced mating [19], place preference as sign of induction of a reward state can only be seen after paced mating [6], [30]. Therefore, we aimed to investigate whether the positive effects of mating, for example on anxiety-related behavior, are only observable under paced mating conditions and whether this is accompanied by release of OT within the brain.

## Results

### Effects of priming, and paced or unpaced mating on anxiety

**Figure 1:**
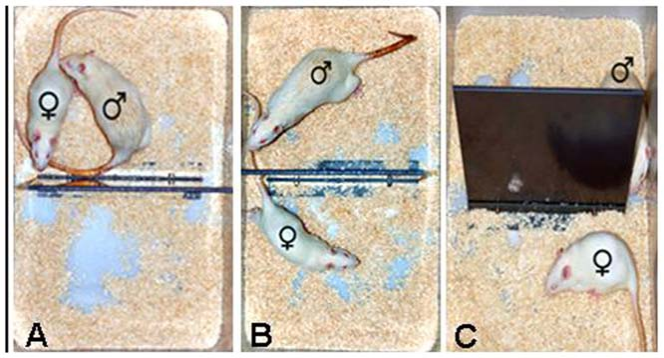
Paced mating (PM) arena. PM was performed in an open-topped PM arena (34×31×53 cm) divided by a vertical barrier with an interspaces of 3.5 cm between the barrier and the cage's walls. The PM arena allows size-dependent withdrawal of the female to the other side of the cage and control of mating frequency and performance of microdialysis during ongoing PM. (A) anogenital investigation of the female by the male, (B) escape of the female to the other side of the barrier, (C) separation of male and female rats.

In order to study the effects of steroid-priming and different mating conditions on anxiety-related behavior in females, ovariectomized non-primed or primed Wistar rats were tested on the elevated plusmaze or in the black-white box [9] 30 min after a 30-min period of single-housing, unpaced or paced mating. Unpaced mating was performed in a standard rat cage. For paced mating, the female and the sexually experienced male were placed in a paced mating arena (Figure 1) allowing the smaller female to escape from the larger male around a dividing wall. Females were repeatedly accustomed to the paced mating arena before the experiment.

There was no significant difference between the number of intromissions during 30 min of unpaced (14.0±3.68) versus paced mating (20.9±2.60; p>0.05). The priming regimen and administration of estrogen and progesterone 48 hrs and 4–6 hrs before mating, respectively, resulted in a prolonged rise in plasma estrogen (F_7,42_6.36, P<0.001) and progesterone (F_1,5_20.84.1, P<0.01) up to 27 hrs after administration (see Figure S1).

**Figure 2:**
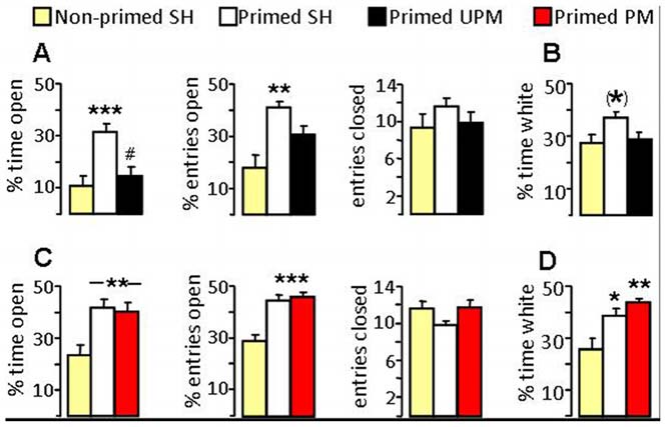
Effects of priming, unpaced (UPM) and paced mating (PM) on anxiety-related behavior. Female, ovariectomized Wistar rats which were either steroid-primed or non-primed were tested on the elevated plusmaze (A, C), or in the black-white box (B, D) 30 min after a 30-min period of single-housing (SH), UPM (A, B) or PM (C, D). Priming-induced anxiolysis remained after PM, but not after UPM, indicated by longer and more frequent exploration of the open and unprotected arms of the plusmaze or the white compartment of the black-white box. Locomotor activity was reflected by the number of closed arm entries on the plusmaze (A, C). Data represent mean + S.E.M. Group size between 9 and 16. *** P<0.001, ** P<0.01, * P<0.05 versus non-primed SH; (*) P = 0.07 versus non-primed SH. # P<0.01 versus primed SH.

In the first experiment, we compared the level of anxiety between non-primed/single-housed, primed/single-housed and primed/unpaced-mated females (Figure 2A, B). The anxiety-related behavior was found to differ between groups both on the plusmaze (factor mating; percentage of time on open arms: F_2,34_10.41, P<0.001; percentage of entries into open arms: F_2,34_11.46, P<0.001; Figure 2A) and in the black-white box (percentage of time in white box: F_2,31_3.33, P = 0.05; Figure 2B) 30 min after unpaced mating. Specifically, priming consistently reduced the level of anxiety both on the plusmaze (P<0.001 and P<0.01, respectively) and in the black-white box (P = 0.07, primed/single-housed versus non-primed/single-housed). However, to our surprise, the anxiolytic effect of priming was almost completely reversed by a 30-min exposure to unpaced mating conditions resulting, for example, in diminished percentage of time spent on the open arms of the plusmaze (P<0.01; Figure 2A). Neither priming nor unpaced mating affected locomotion, as the number of closed arm entries (plusmaze) was similar between groups (Fig 2A).

In the next experiment, we assessed the effect of paced mating on anxiety and compared non-primed/single-housed, primed/single-housed and primed paced-mated female rats (Fig 2C, D). We could reveal significant differences between these groups both on the plusmaze (percentage of time in open arm: F_2,34_ 8.13, P<0.001; percentage of entries into open arms: F_2,34_17.9, P<0.001; Figure 2C) and in the black-white box (percentage of time in white box: F_2,36_7.73, P<0.001, Figure 2D). In contrast to unpaced mating, the low level of anxiety seen after priming remained after paced mating, but paced mating did not further increase the exploration of open arms of the plusmaze (Figure 2C) or the white box (Figure 2D).

In order to further investigate the possible anxiolytic effect of paced mating, we modified the experimental conditions in order to prevent a possible ceiling effect of priming. However, neither (i) more anxiogenic black-white box conditions (increased light intensity in the white box) (Figure S2A) altered anxiety-related behaviour after paced mating nor (ii) prolonged mating duration (60 min instead of 30 min) (Figure S2B), Further, (iii) combining these modified conditions with a prolonged resting period (240 and 180 min respectively) between mating and behavioral testing did not reduce the anxiety-related behavior of paced mating.

Again, the anxiolytic effect of priming itself could be found even up to 180 min after mating independent of the experimental conditions (F_2,16_6.87, P<0.01 Fig S2A), but disappeared after 4 hrs, i.e. 9 hrs after priming (Figure S2B).

### Effects of priming and paced or unpaced mating on central OT release

**Figure 3:**
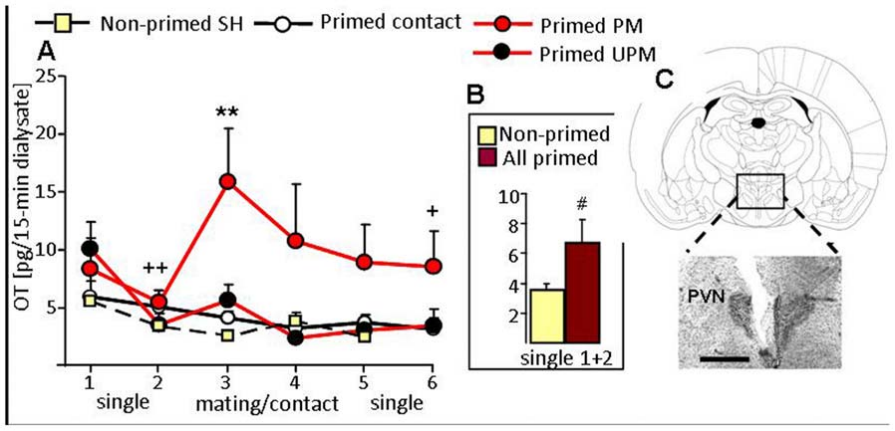
OT release within the PVN during UPM and PM in non-primed and primed female rats. (**A**) PM triggers OT release into the extracellular fluid of the PVN of primed female Wistar rats as indicated by elevated OT content in microdialysates sampled during exposure to a sexually experienced male. Two 15-min microdialysates were sampled during single-housing (dialysate 1, 2), physical contact with an unknown female (social contact), UPM or PM (dialysate 3, 4), and after removal of the female or male (dialysate 5, 6). (**B**) Mean OT content in dialysates sampled from all non-primed or primed females during single-housing (mean samples 1 and 2). (**C**) Schematic drawing at the level of the hypothalamic paraventricular nucleus (PVN; bregma −1.88 mm), and microphotograph of Nissl-stained coronal brain section after removal of the microdialysis probe located inside the PVN. Scale bar, 1.0 mm. Data represent means + S.E.M. ** P<0.01 versus mating/contact all other groups, + P<0.05, ++ P<0.01 versus sample 3, primed PM group; and # P = 0.06 versus mean basal of all non-primed SH females.

In order to reveal differential effects of priming and mating conditions on OT release within the PVN, intracerebral 15-min microdialysates were sampled under basal conditions (samples 1, 2), and subsequently during either single housing, social contact with an unknown ovariectomized female, paced or paced mating (samples 3, 4). Local OT release differed between groups (factor mating x time: F_15,140_ 2.41, P<0.01; Figure 3A). A significant rise in OT release was only found during paced mating compared with basal samples (P<0.01), and with samples collected from non-primed females during single housing, and from primed females during social contact (P<0.01), or during unpaced mating (P = 0.05).

Priming *per se* tended to increase basal OT release within the PVN (P = 0.06 mean basal samples 1 and 2 of all primed (single-housed, unpaced- and paced-mated) groups versus the non-primed single-housed group (Figure 3B).

### Effects of an OT receptor antagonist (OTA)

**Figure 4:**
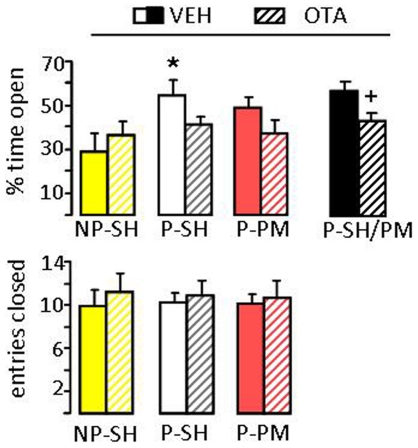
Effects of an oxytocin receptor antagonist (OTA) on anxiety-related behavior in female rats. Female rats were classified into three groups: (i) non-primed single-housed (NP-SH) (ii) primed single-housed (P-SH) and (iii) primed paced-mated (P-PM). NP and P rats were infused with either a selective OTA (0.75 µg/5 µl, intracerebroventricular, hatched bars) or vehicle (plane bars) into the lateral ventricle immediately after 30 min of PM or SH, and 30 min before testing on the plusmaze. A significant effect of OTA on anxiety-related behavior indicated by the percent time spent on the open arms of the maze was found after combining behavioral data from both primed groups (right, black columns). Locomotion reflected by the number of closed arm entries was not altered by any treatment. Data represent means + S.E.M. * P<0.05 versus vehicle-treated non-primed SH females, + P<0.05 versus vehicle-treated groups (combined primed single-housed and paced-mated groups treated with either vehicle or OTA).

As OT exerts a local anxiolytic effect within the PVN [11] and local OT release tended to increase in primed females which reached significance during paced mating (Figure 3), we determined whether central OT is causally involved in low levels of anxiety. We blocked OT receptors which are widely distributed throughout the brain [31] by administration of a selective OT receptor antagonist (OTA) into the lateral ventricle immediately after mating and removal of the male. Thirty min later, we could confirm the anxiolytic effect of priming on the plusmaze in vehicle-treated rats (percentage of time spent on the open arms: F_2,42_ 3.44, P<0.05; Fig 4A). OTA did not significantly alter anxiety-related behavior (factor mating x treatment; F_2,42_ 1.88, P>0.05). However, separate statistics has been performed combining both primed single-housed and primed paced mated groups which were treated with either vehicle or OTA. Comparison of all primed vehicle-treated and all primed OTA-treated groups revealed increased anxiety in OTA-treated females (P<0.05, Mann-Whitney U-test). This indicates that locally released OT appears to contribute to the anxiolytic effect of priming and paced mating. Locomotor activity indicated by the number of closed arm entries was not affected by this treatment, indicating a specific effect on anxiety (Figure 4B).

## Discussion

Our results show that in female rats, control of mating is required to trigger positive consequences on anxiety-related behavior and brain OT system activation. Whereas mating in the absence of female control, i.e. during unpaced mating conditions reversed the priming-induced anxiolysis in female rats in two independent behavioral tests, the emotional responsiveness remained at the low level only when the female could pace the sexual encounter. Moreover, the activation of the brain OT system reflected by elevated OT release within the hypothalamic PVN could only be confirmed in paced-mated, but not in unpaced mated, females. This leads us to the conclusion that sexual activity can have beneficial effects in females comparable to those seen in males during successful mating [9], but this is dependent on the female having control of mating frequency.

Activation of the brain OT system is triggered by various close social interactions, such as, for example, during suckling in the lactating mammal [23] or during sexual activity [18], [19] (Figure 3). In this context, brain OT has been associated with the regulation of these physiological and behavioral functions [32] such as milk ejection, maternal behavior and mother-offspring bonding, penile erection in males [33] and lordosis behavior in females [16] during mating, and pair bonding. In females, high brain OT system activity as seen in the peripartum period [23], [34], [35] has been related to a general reduction in stress responsiveness reflected by attenuated hormonal responses to acute stressors, increased calmness, and reduced anxiety [24]. This finding could be significantly extended in the present study by the demonstration that, in the female rat, priming and paced mating also activate the intrahypothalamic release of OT and consequently, induce anxiolysis. The anxiolytic effects of OT has recently been localized within the central amygdala [10], [35] and the hypothalamic PVN [11]. Within the PVN, such effects are mediated by OT receptors and activation of the intracellular mitogen-activated protein (MAP) kinase pathway [11]. Thus, brain OT activated by close social interactions is an important mediator of the beneficial, mainly stress-protective and rewarding effects of being social on general well being and health [8]. However, in females, the quality of social interactions seems to significantly determine its consequences on emotionality and well being, as shown in the present experiments by the comparison of paced- versus unpaced-mated females.

OT also acts within the nucleus accumbens where it has been related to reinforcement and reward [13], for example, for maintaining pair-bonding in female monogamous voles [36]. In the context of female sexual activity, only paced mating induces a state of reward [6] which depends on interactions between the mesolimbic dopaminergic and the OT systems [37]. Moreover, it has been found both in males and females that the conditioned place preference induced by rewarding mating experiences, e.g. during paced mating in females, involves the activation of the endogenous opioid system [38]. Thus, the possibility exists that the activation of the opioid system contributed to the anxiolytic and rewarding effect of priming/paced mating. This may consequently, explain why administration of the OTA could only partly prevent the anxiolytic and rewarding effects of priming/paced mating. This possibility is substantiated by the profound anxiolytic effect of opioids [39], [40]. Taken together with our present results, it seems likely that only paced mating activates the central OT and reward systems. In contrast, unpaced mating is experienced as being rather stressful and does not stimulate intra-PVN release of OT. Consistently unpaced mating results in an increased level of anxiety compared with single-housed primed controls.

Estrogen and progesterone treatment itself profoundly reduced anxiety both on the plusmaze and in the black-white box confirming recent results [27], [28]. Our finding of elevated intra-PVN OT release after priming under basal conditions (Figure 3B) suggests that high circulating steroids (see Figure S1 and File S1) contribute to elevated OT activation. In support, central OT receptor expression is increased during proestrus [41], and estrogen binds to OT receptors in some limbic and hypothalamic cell groups [32]. In our study, blockade of brain OT receptors by the OTA partly reversed estrogen- and progesterone-induced anxiolysis, indicating that the steroid-induced activation of brain OT contributes to the strong anxiolytic effect of priming.

Reduced state anxiety found in primed females could also be due to the action of progesterone in the ventral tegmental area and other brain regions [42]. Like other neurosteroids, progesterone can exert rapid nongenomic effects on a number of identified neurotransmitter substrates including dopaminergic and GABAergic transmissions [43], [44].

Priming-induced anxiolysis in the female rodent might be essential to allow the approach of the usually larger male. The sustained low level of anxiety found after priming remained after paced mating, but was consistently reversed by unpaced mating. However, we did not find a further reduction in anxiety between 30 and 180 min after paced mating as seen in male rats [9] likely due to a ceiling effect of priming. However, even when behavioral testing was performed under more anxiogenic conditions, for example during increased light intensity in the lit compartment of the black-white box, or allowing a prolonged mating period (Figure S2), anxiolysis directly related to paced mating could not be revealed.

In conclusion, we have shown that only mating under female control increases intra-hypothalamic release of OT which is likely to contribute to the maintenance of the anxiolytic state induced by priming. In contrast, exposure of female rats to unpaced mating fails to induce OT activation and is aversive as rather indicated by increased anxiety. Our results increase our understanding under which conditions brain OT mediates the beneficial consequences of close social interactions on general well being in mammals. Sexual activity has been shown to exert positive health effects in humans and other mammalian species [1]–[5]. Thus, in females, mating-induced consequences on emotional responsiveness strongly depend on the mating conditions and the neuropeptide OT is a possible mediator of the positive effects of sexual activity in females as in males [8]. In as much as the evolutionary advantage of mating-induced anxiolysis is obvious for male mammals, it may be associated in females to calmness and as it is rewarding, increase the urge to search for mating partners and thus facilitate reproduction.

## Materials and Methods

### Ethics statement

All experiments were approved by the local Bavarian government and performed in accordance with the *Guide for the Care and Use of Laboratory Animals* by the National Institute of Health (Permit Number:.54–2531.2–16/08). All surgery was performed under isofluran anesthesia, and all efforts were made to minimize suffering.

### Animals

Sexually naïve adult female (200–250 g body weight) Wistar rats (Charles River, Bad Sulzfeld, Germany) were kept under standard laboratory conditions (12∶12 light/dark cycle, lights off at 10.00 a.m., 22°C, 50% humidity, food and water ad libitum). Female rats were ovariectomized three weeks before the experiments, single-housed for one week and kept in groups of 4 afterwards until 2 days prior to the mating experiments when they were again single-housed. Steroid-primed females received ß-estradiol (200 µg/0.2 ml corn oil, sc) and progesterone (500 µg/0.2 ml corn oil, sc, Fluka Chemie GmbH, Buchs, Switzerland) 48 hrs and 4–6 hrs before start of the mating experiments, respectively. Non-primed rats received 0.2 ml corn oil. Females were submitted to the priming regimen 7 days before the experiments to reduce nonspecific stress. Mating experiments were performed between 12.00 and 03.00 p.m., i.e. 2 to 5 hrs after lights off except stated otherwise.

### Experimental protocols

#### Effects of steroid-priming and paced or unpaced mating on anxiety-related behavior

Ovariectomized females were divided into the following groups: non-primed single-housed, primed single-housed and primed females mated with a sexually experienced male. Mating was performed under either unpaced or paced mating conditions (see below). In preliminary experiments, a social contact group exposed to an unknown female was included but consistently showed no differences to SH rats with respect to anxiety-related behavior.

For unpaced mating experiments, females either remained in their home cage alone (single-housed groups) or were exposed to the male for 30 min without chance to escape the male [45]. For paced mating experiments, the female was placed in the mating arena 30 s before the male was placed on the opposite side of the dividing wall for 30 min (paced mating group), or females stayed alone in the arena for 30 min (single-housed groups). All females were accustomed to the paced mating arena twice for 30 min before the paced mating experiments.

Thirty min (or 180 or 240 min, see Figure S2) after removal of the male (unpaced mating and paced mating experiments) and return of the female to the homecage (paced mating experiments), the female was tested on the elevated plusmaze or black-white box.

In order to exclude a ceiling effect of priming on anxiety, we performed paced mating experiments under more anxiogenic test conditions. Thus, paced mating and control females were tested in the black-white box using a higher light intensity in the white compartment (450–550 lux), respectively.

Another set of females was paced mated at the end of the dark phase (08.00 p.m.) and tested in the black-white box 180 min later during the light phase, i.e. when anxiety levels are elevated and the influence of ovarian hormones on anxiety is low [27].

#### Microdialysis and OT release within the PVN

To study the effects of unpaced mating and paced mating on the activity of the brain OT system, we monitored OT release within the PVN in another set of female rats before, during and after ongoing mating. Two days after stereotaxic implantation of a microdialysis probe into the PVN [46], six consecutive dialysates were collected (see File S1) under basal single-housed-conditions (sample 1, 2 in all groups), during continuing single housing (non-primed females), in the presence of either an unknown ovariectomized female (social group, primed) or a sexually experienced male (mated group, primed) (samples 3, 4) and after removal of the respective animal (single-housed, samples 5, 6). OT content in lyophilized microdialysates was quantified by radioimmunoassay [47].

#### Administration of an oxytocin receptor antagonist (OTA)

In order to investigate the involvement of brain OT in the anxiolytic effect of priming and/or paced mating, stereotaxic surgery for the implantation of the intracerebroventricular (ICV) guide cannula was performed five days before behavioral testings. After a recovery period of five days, a selective OTA (desGly-NH_2_ (9),d(CH_2_)_5_[Tyr(Me)_2_,Thr_4_]OVT; 0.75 µg/5 µl) or vehicle (sterile saline) was infused into the right lateral ventricle through the guide cannula immediately after mating and removal of the male (see File S1). After 30 min, females were tested on the elevated plusmaze for anxiety-related behavior.

#### Paced mating chamber

The paced mating arena for rats has been adapted to be suitable for microdialysis performed during ongoing paced mating. It consists of an open-topped arena (34×31×53 cm) divided into two chambers by a plastic barrier (Figure 1), which allows passage of the smaller female but not the male via a 3.5 cm wide vertical interspaces. This setup simulates a semi-naturalistic condition of PM without spatially confining the male, which may disrupt male sexual behavior [48]. Female rats were habituated to the paced mating arena twice a day for 5 days prior to the experiment to reduce unspecific stress responses.

#### Elevated plusmaze

For quantification of anxiety-related behavior, rats were placed on the elevated plusmaze for 5 min [9]. Increased open arm (140 lux) exploration (percentage of time spent on and number of entries performed into the open arms) reflects reduced anxiety and was scored using a video/computer system. The number of closed arm (20 lux) entries was taken as indication of locomotor activity.

#### Black-white box

In addition to the elevated plusmaze, the black-white box has been employed as another test for anxiety-related behavior [9]. It consisted of a lit compartment (40×50 cm, 350 lux, except stated otherwise) and a black compartment (40×30 cm, 70 lux; opening: 7.5 cm diameter). The rat is placed into the black box, and the percentage of time spent in the white box (anxiety) was scored during a 5-min test period using a video/computer system.

#### Statistics

All statistics were performed using SPSS 18.0 (SPSS Inc., Chicago, USA). Behavioral data were analyzed using a one-way (factor mating) or two-way (factor treatment x mating) analysis of variance (ANOVA); microdialysis results were analyzed using two-way ANOVA for repeated measures (factor mating x time) followed up by Bonferroni *post hoc* tests for pair-wise comparisons, and Mann-Whitney U-test for 2 treatment groups. Data are presented as mean + S.E.M. Significance was accepted at P≤0.05.

## Supporting Information

Figure S1 **Plasma sex-steroid concentrations during the priming and mating regimen.** Ovariectomized female rats were fitted with a chronic jugular vein catheter 6 days prior to priming, and 10 blood samples (0.2 ml) were collected during a 27-h period after treatment. Females received s.c. injections of 200 µg ß-estradiol (E) or oil (VEH) 48 h prior to (A), and 500 µg progesterone (P) or VEH 4–6 hrs (B) prior to mating. Priming increased plasma concentrations of E and P up to 27 hrs later. Data represent means + S.E.M. * P<0.05, ** P<0.01, *** P<0.001 versus VEH treated; + P<0.05 versus time point 4, 6 and 8 h post injection within the E primed group; # P = 0.07 versus VEH treated.
(TIF)

Figure S2 **Effects of priming and paced mating (PM) on anxiety-related behavior under various anxiogenic conditions.** Non-primed and steroid-primed female rats were tested in the black-white box either 240 min after a 30-min PM period using higher light intensity (450–550 lux) in the white box (A), or 180 min after prolonged PM (60 min, B) in the light phase or after single-housing (SH). Priming and PM resulted in reduced anxiety 240 min (A, n.s.) and 180 min (B; P<0.05) after mating, but a further anxiolytic effect of PM itself was not found thus confirming data in Fig 2. The percentage of time spent in the white box indicates anxiety-related behavior. Data represents mean + S.E.M. Group size between 5 and 14. ** P<0.01, * P<0.05 versus non-primed SH.
(TIF)

File S1 **Supplemental methods.**
(DOC)
