# Supplementary figures and images for: Yes, I Am Ready Now: Differential Effects of Paced versus Unpaced Mating on Anxiety and Central Oxytocin Release in Female Rats
Source: PLoS One. 2011 Aug 16;6(8):e23599. doi: 10.1371/journal.pone.0023599 (PMC3156771; doi:10.1371/journal.pone.0023599)

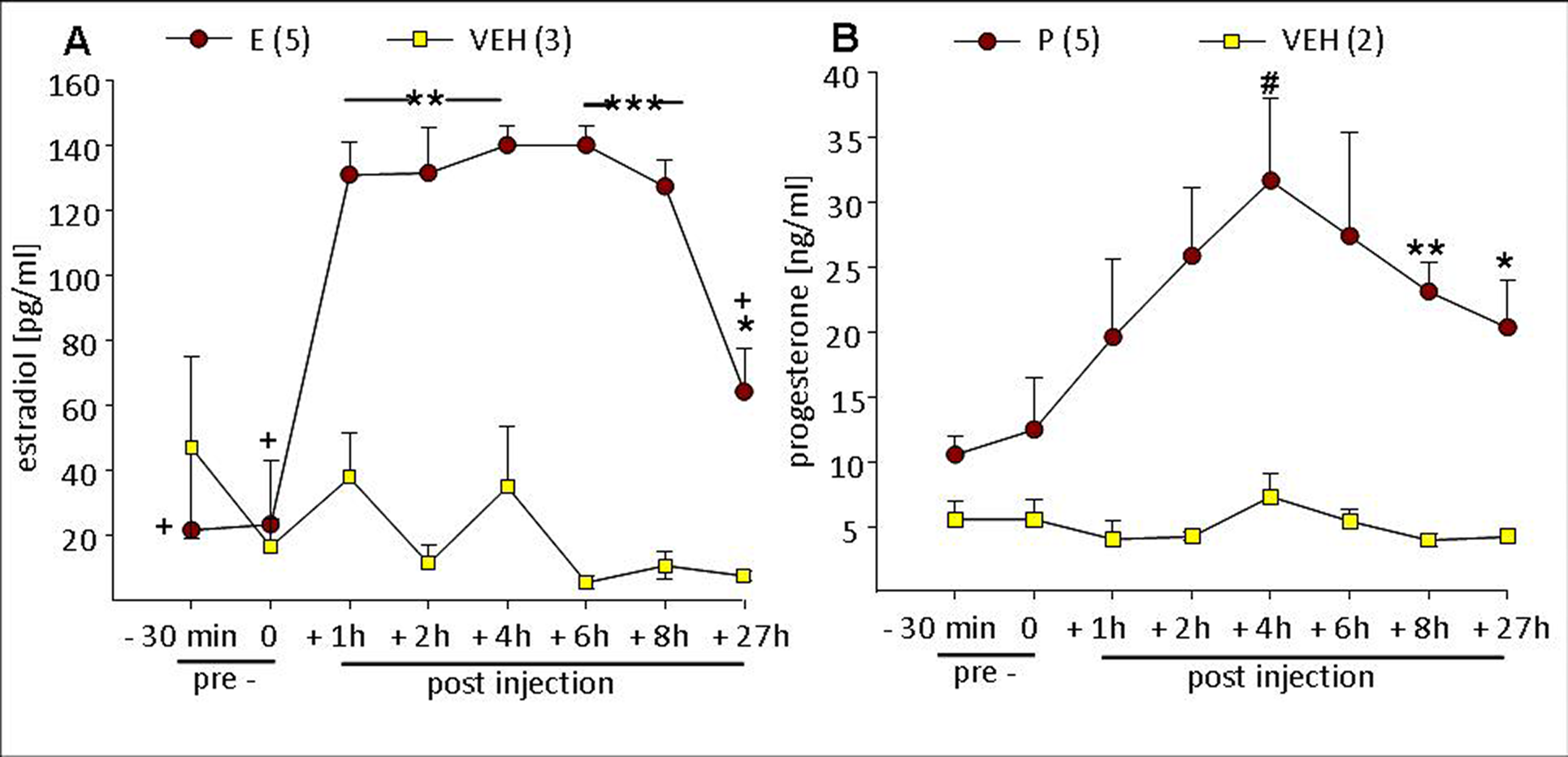

Supplement: Figure S1 — Plasma sex-steroid concentrations during the priming and mating regimen. Ovariectomized female rats were fitted with a chronic jugular vein catheter 6 days prior to priming, and 10 blood samples (0.2 ml) were collected during a 27-h period after treatment. Females received s.c. injections of 200 µg ß-estradiol (E) or oil (VEH) 48 h prior to (A), and 500 µg progesterone (P) or VEH 4–6 hrs (B) prior to mating. Priming increased plasma concentrations of E and P up to 27 hrs later. Data represent means + S.E.M. * P<0.05, ** P<0.01, *** P<0.001 versus VEH treated; + P<0.05 versus time point 4, 6 and 8 h post injection within the E primed group; # P = 0.07 versus VEH treated. (TIF) [file pone.0023599.s001.tif]

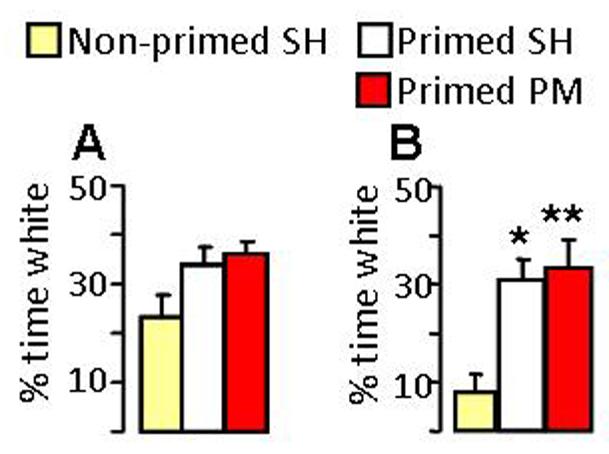

Supplement: Figure S2 — Effects of priming and paced mating (PM) on anxiety-related behavior under various anxiogenic conditions. Non-primed and steroid-primed female rats were tested in the black-white box either 240 min after a 30-min PM period using higher light intensity (450–550 lux) in the white box (A), or 180 min after prolonged PM (60 min, B) in the light phase or after single-housing (SH). Priming and PM resulted in reduced anxiety 240 min (A, n.s.) and 180 min (B; P<0.05) after mating, but a further anxiolytic effect of PM itself was not found thus confirming data in Fig 2. The percentage of time spent in the white box indicates anxiety-related behavior. Data represents mean + S.E.M. Group size between 5 and 14. ** P<0.01, * P<0.05 versus non-primed SH. (TIF) [file pone.0023599.s002.tif]
