# Supplementary material for: Yes, I Am Ready Now: Differential Effects of Paced versus Unpaced Mating on Anxiety and Central Oxytocin Release in Female Rats
Source: PLoS One. 2011 Aug 16;6(8):e23599. doi: 10.1371/journal.pone.0023599 (PMC3156771; doi:10.1371/journal.pone.0023599)
Supplement: File S1 — Supplemental methods. (DOC) [file pone.0023599.s003.doc]

**Supplementary information**

**Yes, I am Ready Now: Differential Effects of Paced Versus Unpaced Mating on Anxiety and Central Oxytocin Release in Female Rats**

Nyuyki et al

Files in this Data Supplement**:**

File S1

**File S1**

**Surgical Procedures**. All surgical procedures were performed under isofluran anesthesia and semiaseptic conditions.

**Jugular vein catheterization**. This was performed as described by Neumann and colleagues [1]. Briefly, the jugular vein was exposed by blunt dissection and the catheter consisting of a silicone tubing (Dow Corning Corp., Midland MI) connected to a polyethylene (PE-50) tubing was inserted approximately 3 cm into the vessel until the tip reached the right atrium. It was then exteriorized dorsally between the ears. The exteriorized PE tip was tightly folded to block the passage of air into the heart. Animals received saline containing gentamicin (30 000 IU/ ml, Centravet, Bad Bentheim, Gemany) after jugular vein catheterization.

For monitoring OT release within the PVN, we implanted a U-shaped microdialysis probe (dialysis membrane: molecular cut-off of 18 kDa; Haemophan; Gambro Dialysatoren, Hechingen, Germany) into the right PVN (1.4 mm caudal to bregma, 1.6 mm lateral to midline, 8.6 mm beneath the surface of the skull and placed at an angle of 10° to avoid sagittal sinus damage [2]) as described before [3].

For icv infusion of OTA, a guide cannula (21 G, 12 mm long) was implanted 2 mm above the right lateral ventricle (1.0 mm caudal to bregma, 1.6 mm lateral to midline, and 1.8 mm beneath the surface of the skull). Both the microdialysis probe and icv guide cannulas were secured in place by 2 stainless screws and dental cement; the icv guide cannula was closed with a stylet. Animals received the antibiotics Baytril (30 µl; Bayer Vital GmbH, Leverkusen, Germany) before stereotaxic surgery to prevent bacterial infection and were kept individually in transparent polycarbonate cages.

To verify the exact placement of the MD probe and the icv guide cannula, 40-µm cryocut slices were stained by Nissl-staining and by icv infusion of Evans Blue dye (5 µl), respectively.

**Blood sampling**. In the morning of the 5th day after surgery, the catheter was connected to an extension PE-50 tube attached to a 1-ml plastic syringe filled with heparinized sterile saline (30 000 IU ⁄ ml, Heparin-Natrium, Ratiopharm, Ulm, Germany). About 2 h later, 2 basal blood samples (0.2 ml) were collected within a 30-min interval. Estradiol (E: 200 µg/0.2 ml corn oil) or corn oil (0.2 ml, vehicle) was administered subcutaneously immediately after the second basal sample was taken. Subsequent blood samples were collected 1, 2, 4, 6, 8 and 27 h after last injection. The catheter was flushed with heparinized saline, the extension tubing disconnected and the catheter closed. In the morning of the 7^th^ day after surgery, the catheters were reconnected and further blood samples were collected as described above, but the animals were treated with progesterone (P: 500 µg/0.2 ml) or vehicle. Withdrawn blood was always replaced by sterilized saline. Plasma E and P were quantified using ELISA (DRG Diagnostics GmbH, Marburg, Germany).

**Intracerebral microdialysis.** Fifteen-minute microdialysates were sampled at 4.5 µl / min (sterile Ringer’s solution, pH 7.4) into 1.5-ml into eppendorf tubes containing 10 µl of 0.1 N HCl and immediately stored at -80 °C. Before the first sample was collected, the microdialysate probe was flushed for 2 h. Oxytocin content in sampled microdialysates was quantified using radioimmunoassay; the detection limit was in the range of 0.1 pg / sample. The intra-assay coefficient of variation at relevant concentrations of the standard curve was between 7 and 10% and there was no inter-assay variation as all dialysates were measured in the same assay [4].

**Intracerebroventricular (ICV) infusion and OTA** **treatment**. Five days after stereotaxic surgery of the icv guide cannula, an infusion cannula (27 G, 15 mm long) was attached to an extension tubing (PE-50, 70 cm) connected to a 50-ul microsyringe. The infusion cannula was inserted into the guide cannula thus reaching the right cerebral ventricle, and OTA (0.75 µg/5 µL; desGly-NH_2_(9),d(CH_2_)_5_ [Tyr(Me)_2_,Thr_4_]OVT) or sterile saline (5 µL 0.9 % NaCl) was slowly infused. The injection system was withdrawn 30 s after infusion in order to allow for proper diffusion.

**References**

1. Neumann ID, Johnstone HA, Hatzinger M, Liebsch G, Shipston M, et al. (1998) Attenuated neuroendocrine responses to emotional and physical stressors in pregnant rats involve adenohypophysial changes. *J Physiol* 508 ( Pt 1):289-300

2. Paxinos G, Watson C, *The rat brain in stereotaxic coordinates*. 4 ed. 1998, Sydney: Academic.

3. Neumann I, Russell JA, Landgraf R (1993) Oxytocin and vasopressin release within the supraoptic and paraventricular nuclei of pregnant, parturient and lactating rats: a microdialysis study. *Neuroscience* 53:65-75

4. Waldherr M, Neumann ID (2007) Centrally released oxytocin mediates mating-induced anxiolysis in male rats. *Proc Natl Acad Sci U S A* 104:16681-4
